# Informing patients on planned consultation time – a randomised controlled intervention study of consultation time in primary care

**DOI:** 10.1080/02813432.2019.1663581

**Published:** 2019-09-09

**Authors:** Oskar Lindfors, Sara Holmberg, Mattias Rööst

**Affiliations:** a AMK Kronoberg, Kronoberg County Centre for Competence in Primary Health Care, Kronoberg, Sweden;; b Department of Research and Development, Växjö, Sweden;; c Division of Occupational and Environmental Medicine, Institute of Laboratory Medicine, Lund University, Lund, Sweden;; d Department of Clinical Sciences, General Practice/Family Medicine, Lund University, Malmö, Sweden

**Keywords:** Mesh terms, primary health care, general practice, appointments and schedule, physician patient relations, patient centred care, patient satisfaction

## Abstract

**Objective:** To investigate whether patients’ pre-consultation knowledge of the time frames for the consultation influences the actual consultation time and/or patient and physician related outcomes; satisfaction and enablement.

**Design:** Randomised controlled blinded intervention study.

**Setting:** Four strategically chosen Primary Health Care Centres (PHCC:s) in Kronoberg county in Sweden participated.

**Intervention:** Pre-consultation information on planned consultation time. During one week in each PHCC consecutive patients were randomised to intervention group or control group, when booking an appointment with a physician.

**Subjects:** Patients >18 years of age.

**Main outcome measures:** Consultation time, patient satisfaction, patient enablement and physician satisfaction.

**Results:** No significant difference in consultation time was found between the intervention group and control group. No differences were seen between intervention group and control group regarding any of the other measures. Stratified data showed significantly shorter consultation time for the intervention group in one of the PHCC:s and for employed physicians. Employed physicians also rated consultations as being easier and were more satisfied with their consultations compared to non-employed physicians.

**Conclusion:** Information on the planned consultation time has a potential to decrease consultation time in certain settings. No negative side effects were found in this study.

**Key points**
- Patients prepare before their consultation but to influence its contents and length is difficult.
- Informing patients on estimated consultation time can influence actual consultation time.
- Informing patients on planned consultation time has no adverse effects in this study.

## Introduction

A minute can be long and a minute can be short. Everyone is familiar with the experience of time and that it can alter depending on the situation [1]. In primary care consultations, time is regarded a crucial resource. Physicians often seem to experience lack of time; a common complaint is difficulties to keep the time schedule and that patients can be difficult to restrain both concerning time and to focus on the issues planned for when booking the appointment [2]. Patients, on the other hand, often seem to want more time or feel that the doctor did not have enough time to listen to them [3]. Time has also been suggested to be a pseudo marker for satisfaction [4]; physicians skilled in consultation technique might make the patients feel that enough time is offered even though the actual time spent is the same or shorter compared to consultations with less skilled physicians [4–6].

Consultation length in primary health care has been subject to some scientific evaluation [5,7–11]. The results are somewhat contradictory, some studies suggesting that increasing length leads to greater patient satisfaction [12] and others pointing out empathy, communication skills and patient centeredness as more important factors both regarding patient satisfaction and clinical outcomes [13–17]. Further, satisfied patients experience longer duration of the consultation even though the actual time spent was not longer [5].

Various interventions to influence consultation time and/or contents of consultations have been studied, aiming both at changing the actual consultation time, to encourage patients to take a more active role in the consultation as well as to improve physicians’ consultation skills. Though small and/or non-significant changes in the parameters measured often was reported [13,18–21]. Bias and uncontrolled confounding often limits validity; randomisation, control groups and blinding as well as validated instruments are rare, which makes evaluation of effects is difficult [13,20,21].

In an often used model, developed by Risör and Larsen to teach consultation skills, it is suggested that the consultation process starts already before the actual meeting takes place; patients plan what to say and how to deliver their story to the physician beforehand – possibly already before booking the appointment [22–24]. Prior studies trying to impact patients’ consultation behaviour, for example encouraging patients to ask more questions during the consultation, have mostly consisted of ‘waiting room interventions’ [20,21]. It is conceivable that an intervention at this stage is too late to have a considerable influence on patient behaviour in the consultation, as patients according to Risör and Larsen’s model prepare themselves before entering the waiting room. It might even be possible that a waiting room approach could be a disturbance to the consultation process, if patients must concentrate on other things than their own presentation of their problem(s) and are distracted and forced to rethink their plan directly before the consultation starts.

In line with Risör and Larsen’s model, it is reasonable to expect that if patients gain knowledge of the actual time frames of the consultation beforehand this could have an impact on patients’ preparations for the meeting making time frames easier to keep and resulting in more satisfied patients. Studies investigating if knowledge of time frames for a meeting has an impact on actual meeting time and/or outcomes of meetings are scarce in the field of medical science and other disciplines – despite librarian assisted search no studies addressing this topic could be found.

The aim of this study was to investigate whether providing patients with information on the time frames of a planned consultation, thereby giving patients a possibility to incorporate time frames in their preparations, have an impact on actual consultation length and to evaluate if patient satisfaction, patient enablement and/or physicians’ experiences were affected by this information.

## Methods

### Design

Randomised controlled blinded intervention study.

### Setting

The study was conducted at four strategically chosen publicly financed Primary Health Care Centres (PHCC:s) in Kronoberg County in southern Sweden. The strategic choice was based on aspects such as feasibility of the study, staffing situation, number of registered patients and socioeconomic differences in the populations, aiming at retrieving representative data for the population in Kronoberg County and in Sweden.

One PHCC was situated in a small municipality, one in the County main town, one in a small town and one on the countryside. The number of citizens registered at each unit varied from 4700 to 11,000. Data collection was performed during one week at each PHCC during autumn 2016 and spring 2017.

Often in Sweden, appointments at PHCC:s are booked after a phone consultation with a nurse or by letter for scheduled medical check-ups. When booked for an appointment with a physician, patients normally get information about the starting time for the appointment but in most cases the time frames for the consultation are not described to the patient. However, it is presumptive that a majority of patients would understand that a time limitation exists. Planned booking intervals, appointment modules, can vary but in the studied PHCC:s 15 min and 30 min modules were most common.

Lack of physicians in primary care is frequent in Sweden and short term temporary physicians, locums, are common. In this study locums along with post graduate residents are referred to as ‘non-employed’ whereas General Practioners (GP:s), GP trainees and other long term employed physicians are referred to as ‘employed’.

### Study population

Consecutive patients aged 18 years and above booked for an appointment with a physician were included for randomisation. Exclusion criteria were poor knowledge of Swedish and cognitive impairment, as judged by the booking nurse before randomisation or by the physician during the consultation.

### Main outcome and study variables

Consultation time, measured in whole minutes, was the main outcome. Starting time and ending time of each consultation was noted by the physician using a digital clock in the reception room.

Questionnaires were used to assess patient and physician related outcomes. The patient enablement instrument (PEI) [25] and part of the EUROPEP – instrument [26] concerning ‘satisfaction with your physician’ were used in the patient questionnaire.

PEI is a six item questionnaire that has been translated to and validated in Swedish [27]. The instrument focuses on the impact of consultations on patients’ self-perceived ability to understand and cope with health issues and disease. It is described to be related to, but different from, measures of satisfaction. Each item has three ordinal options that give 0, 1 or 2 points and a ‘not relevant’ option that gives 0 points. The items are summed up to a total score that can vary from 0 to 12 points [25].

EUROPEP is a patient satisfaction instrument regarding different aspects of the medical care and the provided medical service as a whole [26]. EUROPEP has been translated to Swedish even though the Swedish version has not been validated. The part of EUROPEP used in this study consists of six items, each with five ordinal options that gives 1–5 points. A ‘not relevant’ option was added as some items might not feel relevant when the questionnaire was applied to one specific consultation rather than evaluating the medical care as a whole. The items were summed up to a total score, which range from 6 to 30 points.

If one or more items in either of the two instruments were not filled out (missing) or if the ‘not relevant’ option in the EUROPEP was chosen that instrument was excluded from the analyses for that patient.

The physician questionnaire was developed by the authors and in addition to starting and ending time as described above, consisted of five statements with four ordinal response options as to which degree the physician agreed to the statement. The statements covered how easy the physician found the consultation, if the patient was structured regarding the anamnesis, if the anamnesis was fitted to the time frames, if the physician believed that the patient was satisfied with the consultation and if the physician was satisfied. Further, the physician noted which appointment module the patient was booked for, if any exclusion criteria were fulfilled and finally two control questions to ensure blinding. The physician questionnaires were pre-marked with the physicians’ initials and data were collected regarding sex and employment situation for each physician.

### Intervention

The intervention consisted of information on the planned duration of the consultation, in addition to the starting time. The control group was given information on the starting time only, in accordance to usual care.

### Procedure

Excel^®^ was used to make pre-randomised numbered lists. During the booking procedure, which was carried out by a booking nurse over the phone or by letter, the patient was assigned the first available number from the randomised number list.

The booking nurse informed the patient about the starting time (both groups) and the planned duration of the consultation (intervention group only). Consensus on time frames for the patient information in the intervention group was reached through discussions within the research group and other colleagues. For 30 min appointment modules the time frame was set to 20 min, for 20 min modules to15 min, for 15 min modules to 10 min and for 45 min modules to 30 min. These estimations of planned consultation time correlates well to a prior study on mean consultation length (actual face to face time) in Sweden [8].

At arrival at the PHCC the patient was asked to participate by the receptionist who also gave the patient oral and written information about the study. After the consultation the physician handed out the patient questionnaire. The physician also completed the physician questionnaire and filed this during the study week.

The physician was blinded to which group the patient belonged to. Patients were blinded to the fact that two groups existed and that difference in time and results from the questionnaires between the two groups was studied. The study information only contained information about that the consultation time was measured and that a questionnaire about the meeting should be filled out.

### Statistical analyses

A power calculation was made using data from a pilot test which was carried out in one of the four PHCC:s. An estimate of the needed sample size gave 124 subjects per group with a statistical power of 0.8 with a 1.5 min time difference (SD 4.2 min) between the groups. A 1.5 min difference was considered by the authors to be needed to have a clinical relevance.

IBM SPSS^®^ ver. 23 was used for statistical analyses. Background data was compared with two sided *t*-test for age and with Chi^2^-test for the other variables. Mean time was compared with two sided *t*-test between groups. PEI- and EUROPEP-scores as well as the questions from the physician questionnaire were analysed using Mann–Whitney’s test.

### Ethical considerations

The study was approved by the regional ethical committee in Linköping, J no 2017/15-31.

## Results

**Figure 1.**
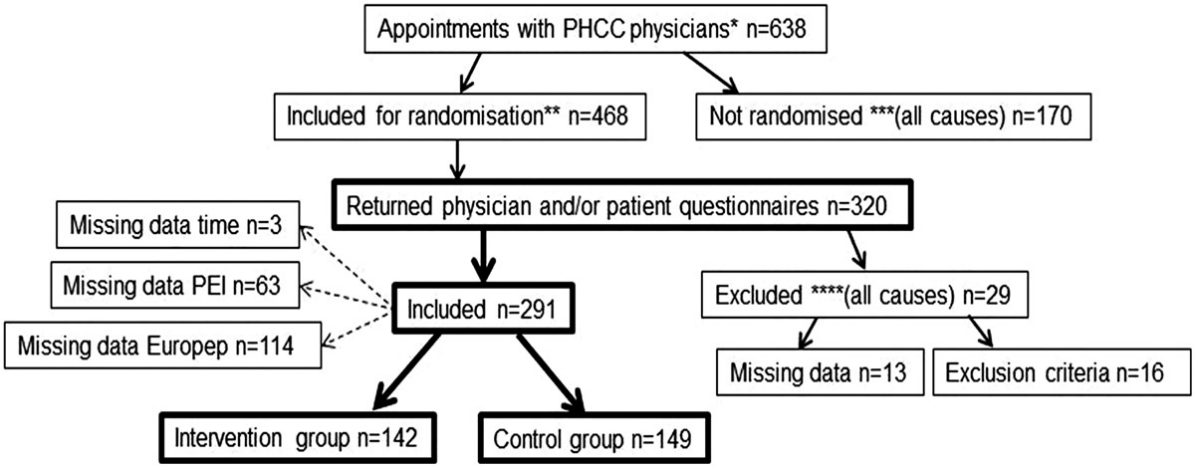
Flow chart of inclusion and randomisation process. *All patients booked for an appointment with a physician at the four PHCC:s during the study. **Patients >18 years old, were included to be randomised during the booking of an appointment to a physician. ***Patients <18 years old, not speaking Swedish, in need of an interpreter or had a known cognitive impairment were not included to be randomised. ****Patients that spoke too little Swedish or had a cognitive impairment, as judged by the physician in the physician questionnaire, were excluded as well as patients where the individual study number was confused, forgotten by the physician or mistakenly used in duplicate questionnaires.

The inclusion process is described in Figure 1. Totally 291 patients were included, 142 in the intervention group. Mean age was 57.3 years (SD = 19.8) and a majority of patients were women (64.6%). There were no differences between the intervention and the control group regarding background data except for 30 min appointment modules. The majority of consultations were booked for 30 min appointment modules (69.1%) and for those, more patients were randomised to the control group than to the intervention group (75.8% vs. 62.0%, *p* = 0.03). Characteristics of control and intervention groups are shown in Table 1.

**Table 1.** Background characteristics.

	Intervention group	Control group
Mean age years (range)	56 (18–90)	58 (19–94)
Sex patients, *n* (%)		
Female	72 (50.7)	72 (48.3)
Male	44 (31.0)	35 (23.5)
Missing	26 (18.3)	42 (28.2)
Sex physician, *n* (%)		
Female	47 (33.1)	53 (35.6)
Male	95 (66.9)	96 (64.4)
Physician employment^a^, *n* (%)		
Employed	77 (54.2)	90 (60.4)
Non-employed	65 (45.8)	59 (39.6)
Appointment module^b^, *n* (%)		
30 min	88 (62.0)	113 (75.8)
15 min	20 (14.1)	11 (7.4)
Other	19 (13.3)	18 (12.2
Missing	15 (10.6)	7 (4.7)

^a^Long term temporary physicians, trainees in general practice and employed General Practitioners were categorised as Employed whereas locums, other short term temporary physicians and post-graduate residents were categorised as Non-employed.

^b^Time frame in the physician schedule which includes the consultation and administrative duties related to the appointment. The planned consultation time informed to patients was shorter, see methods section.

Mean consultation time for all included patients was 19.1 min (SD 8.2), with no significant difference between the intervention and the control group (Table 2). In one PHCC the consultation time in the intervention group was significantly shorter (17.0 min vs. 21.7 min, *p* = 0.006).

**Table 2.** Results mean consultation time in the intervention group compared to the control group for all appointments, for appointments in the most common appointment modules and stratified on physician employment situation. Bold values are significant *p* values.

	Intervention group		Control group		
Mean time (min)	Time (min)	*n*	Time (min)	*n*	p*
Total	18.4	141	19.7	147	0.19
30 min appointment module	19.3	87	20.9	113	0.17
15 min appointment module	14.3	20	14.6	11	0.86
Mean time employees (min)					
Total	17.6	76	20.3	89	**0.04**
30 min appointment module	18.6	51	22.0	71	**0.02**
15 min appointment module	13.9	11	15.0	4	0.74
Mean time non-employees (min)					
Total	19.4	65	18.7	58	0.66
30 min appointment module	20.4	36	19.1	42	0.48
15 min appointment module	14.8	9	14.4	7	0.88

*p was tested using two-sided *t*-test.

Median PEI score was 3.5 (IQR 1–6) and median EUROPEP score was 28 (IQR 25–30), there were no significant differences between the intervention and control groups (PEI score 4, IQR 1-–6 vs. PEI score 3, IQR 0–6, *p* = 0.36. EUROPEP score 28, IQR 26–29, vs. EUROPEP score 27.5, IQR 24–30, *p* = 0.36).

No significant differences were found between intervention and control groups for any of the items in the physician questionnaire.

Stratified data for physicians’ employment situation showed significantly shorter consultation time in the intervention group compared to the control group for employed physicians (17.6 vs. 20.3 min, *p* = 0.04). Employed physicians more often found the consultation to be easy (Q1, 81.2% vs. 67.7%, *p* = 0.01) and more often were satisfied with the consultation (Q5, 86.1% vs. 75.8%, *p* = 0.03) compared to non-employees.

## Discussion

The results do not imply a general impact on consultation time in the intervention group but had effect within certain conditions. Employed physicians had shorter consultation time in the intervention group compared to the control group and they also found consultations in both intervention and control group easier and were more satisfied with their consultations compared to non-employees.

The main strength of this study is its design that included blinding and randomisation, techniques that have been rarely used in studies about primary care consultations [13,20,21]. The randomisation process was controlled by two questions in the physician questionnaire that did not imply that there were any problems with the blinding. Validated questionnaires for patient related outcomes were used. We regarded EUROPEP as the most feasible measure of patient satisfaction for this study due to the existing translated version and the comprehensiveness of the questions. The intervention did not show any effect on patient satisfaction. However, as patients generally report high degrees of satisfaction with care providers, comparisons of such outcomes are difficult. Two other instruments, the Medical Interview Satisfaction Scale (miss-21) and the Consultation Satisfaction Questionnaire eight question version (CSQ-8), that were developed for evaluating a single consultation were considered. Use of a different instrument could have provided other results. However, none of the instruments are validated in the Swedish version and the EUROPEP was evaluated to be most suitable by the authors [28–30].

PEI on the other hand offers a possibility to look beyond satisfaction as it aims at investigating patients’ ability to cope with life, illness and symptoms.

Approximations were necessary to make the study feasible, possibly limiting the accuracy of the results. For feasibility reasons the physicians noted the starting and ending time, even though a more objective method such as recording of consultations and measuring of time afterwards could have been more accurate [11,18]. As we used a patient randomised and a patient and physician blinded design the possible error would be expected to be of the same size in both the intervention and the control group, balancing out the negative effects on accuracy.

The appointment modules in the physicians’ schedules consist not only of the patient consultations, but also of different administrative duties correlated to the consultations. Therefore the planned consultation time has to be considerably shorter than the time available for the physician in the schedule. The estimation of planned consultation time can be further discussed regarding how much time that should be allocated to administrative duties, but was carried out as described in the methods section. As noted in the methods section, it is possible that patients have a predefined idea about face-to face consultation length but more uncertain if they correctly assess the time needed for administrative duties. However, in PHCCs where the study was performed time frames are normally not given to patients.

Time for examinations made by other professions while ‘pausing’ the consultation, such as ECG or blood samples, was not included. For practical reasons and to avoid the risk of missing data (physicians forgetting to fill out the form correctly) for a continued part of the consultation after such examinations, the time for divided consultations with an examination from a person with another profession in-between was noted only for the first part. The hypothesis behind the intervention was that it might address patients’ planning by making it possible to incorporate time in preparations for the consultation and this would more likely affect the first phase, the so-called patient phase, of the consultation. Hence it was considered most important to measure the time for the first part of the consultation [23,24].

The number of included patients was well according to the power calculation. Though, the pilot study on which the power calculation was based was carried out in the PHCC where a significant difference in time between interventions and controls was found in the study. It is possible that specific prerequisites in this PHCC affected the power calculation to underestimate the number of needed subjects.

Not randomising PHCC:s in the study might affect the applicability of the results. Possibly, the employment rate was a bit higher at the selected PHCC:s as it would have been difficult to carry out the study in PHCC:s with many vacancies. All of the studied PHCC:s did have at least one non-employee physician during the study week and in one PHCC the vast majority of consultations included were carried out by non-employees. Although the main reason for engaging the selected PHCC:s was not employment rate of physicians but if the PHCC was well functioning regarding aspects of other professions, as the major part of work for carrying out the study would involve other staff members than physicians.

An interesting finding in this study is the observed difference between employees and non-employees. As the results were somewhat contradictory – consultation time was significantly shorter in the intervention group in one of the four PHCC:s, there was tendency to opposite result in one other PHCC, and as employment rate was one possible explanatory factor for the differences between the units – we collected data on employment situation for each physician and made further statistical analyses. As this study was not designed to explain these findings or to investigate employment situation, the results should be regarded as hypothesis generating. Still, possible explanatory factors might be non-employees getting other types of appointments booked, non-employees being prone to consult in a different way but perhaps most important that employed physicians have a better possibility for continuity of care and to know their patients, which might also explain why they found consultations easier and were more satisfied [12]. In Sweden there is a harsh debate regarding locums, both within the profession and politically, why this finding is of interest for further investigation.

This study investigated if knowledge of time frames for the consultation had an impact on consultation length, theoretically by influencing patients’ preparations. Whether knowledge of time frames for a meeting affects the actual meeting time seem to be an area of research that has been neglected not only in the medical field, which enhances the relevance of this study.

Research on consultations most often have focused on time, contents, outcomes and to some degree the consultation process but little is known when it comes to how patients prepare for the consultation. More research on different consultation entities would be desirable as it is reasonable to expect that high quality consultations would result in better medical and patient outcomes.

Understanding patients’ preparations could enable possibilities to provide patients with tools to improve and strengthen their part in the consultation process and thereby medical outcomes, patient satisfaction and patient enablement. With an increasing work load and a political will to extend the domains and responsibilities for primary care, more knowledge on patients’ preparations for consultations could be one way of making consultations more effective, thus improving working environment for physicians, generating better outcomes and decreasing consultation time. Further studies approaching these questions and how patients perceive getting information on time frames, using both qualitative and quantitative methods are planned to be undertaken by the authors.
